# The financial impact of MD-PhD training compared with MD training for academic physicians

**DOI:** 10.1172/jci.insight.183476

**Published:** 2024-12-20

**Authors:** Eva Catenaccio, Jonathan Rochlin, Myles H. Akabas, Lawrence F. Brass, Harold K. Simon

**Affiliations:** 1 Department of Neurology, Division of Pediatric Neurology, Perelman School of Medicine at the University of Pennsylvania and the Children’s Hospital of Philadelphia, Philadelphia, Pennsylvania, USA.; 2 Department of Emergency Medicine, Division of Pediatric Emergency Medicine, Maimonides Medical Center, Brooklyn, New York, USA.; 3 Departments of Neuroscience and Medicine, Albert Einstein College of Medicine, Bronx, New York, USA.; 4 Department of Medicine, Perelman School of Medicine at the University of Pennsylvania, Philadelphia, Pennsylvania, USA.; 5 Departments of Pediatrics and Emergency Medicine, Emory University School of Medicine and Children’s Healthcare of Atlanta, Atlanta, Georgia, USA.

## Abstract

To reduce debt burden and encourage the pursuit of research-focused careers, most MD-PhD programs provide medical school tuition remission and an annual stipend. However, prolonged training compared with MD physicians postpones the time until MD-PhD physicians earn a full salary. We compared lifetime earning potential for MD-PhD physicians in academia with their MD colleagues in the same clinical specialty. We examined the relationship between earning potential based on specialty and the likelihood that MD-PhD physicians reported being engaged predominantly in research. Lifetime earning potential was estimated using 2020–2021 debt and compensation data for 77,701 academic physicians across 47 specialties. Self-reported research effort for 3,025 MD-PhD program alumni in academia was taken from the National MD-PhD Program Outcomes Study. We found that (a) MD-PhD physicians had a lower lifetime earning potential than MD physicians in the same specialty; (b) there was an inverse relationship between earning potential and research effort in different specialties, with MD-PhD physicians in high-earning specialties tending to spend less time on research; and (c) despite this, MD-PhD physicians in academia were more likely to choose clinical fields that allow more time for research.

## Abstract 2

This study compared lifetime earning potential for MD and MD-PhD physicians across adult medical, adult surgical, and pediatric medical specialties.

## Introduction

The combined MD-PhD training pathway started in the 1950s and expanded with the development of the NIH-supported Medical Scientist Training Program (MSTP) in 1964. As of 2024, more than 15,000 physician-scientists have been trained through the MD-PhD pathway. There are currently over 90 active MD-PhD programs in the United States, of which more than 50 are MSTP supported (1). Although the numbers of MD-PhD programs and of students trained have increased over time, the quantity of graduates produced each year is still substantially smaller than the estimated number needed to sustain the physician-scientist workforce (2). There are multiple reasons for this shortfall, including the demands of the job, the increasingly long training period, and, for institutions, the high cost of training MD-PhD physicians (3).

Supporting MD-PhD training is expensive, both for the universities that host the programs and for the NIH, which defrays the costs of training programs but does not fully cover them. From a purely economic perspective, the extended training duration for future physician-scientists in MD-PhD programs means a delay before a full salary can be earned. Even though MD-PhD program trainees typically receive medical school tuition remission and an annual stipend, savings that amount to hundreds of thousands of dollars, the protracted training duration affects their lifetime earning potential, as do the choices that they make regarding which clinical field to specialize in and how much time to devote to research versus clinical care. Thus, despite tuition remission and stipends, one potential consequence of MD-PhD physicians choosing a research-focused career in academia is a lower lifetime earning potential compared with MD-trained colleagues in the same clinical discipline who choose to focus on clinical care.

We have previously demonstrated wide variation in earning potential between specialties in both pediatric and adult medicine as well as between women and men in academic medicine and have shown that these disparities are associated with workforce distribution (4–9). In this study, we asked (a) whether there is a substantial gap in lifetime earning potential for MD-PhD physician-scientists in academia compared with their MD-trained colleagues in academia in the same specialty and (b) whether the potential gap in lifetime earning potential is associated with choice of clinical discipline and research effort in academia. The results show that the temporary financial advantage afforded by MD-PhD training programs is offset by a reduction in lifetime earning potential, especially in higher earning specialties. Despite this, the data also show that most MD-PhD alumni choose clinical fields that allow more time for research.

## Results

We analyzed salary data representing 77,701 academic physicians from 47 specialties across adult medicine, adult surgery, and pediatric medicine. We employed a net present value (NPV) analysis to estimate lifetime earning potential, which we termed Lifetime NPV, for graduates of MD programs compared with graduates of MD-PhD programs. NPV analysis is a standard financial technique used to evaluate potential investments (10). It addresses the fact that income obtained today is more valuable than future income, and it allows us to estimate the financial returns that an incoming medical student might expect from pursuing a career as an MD or as an MD-PhD across different specialties. Overall, we found that MD-PhD program graduates had lower Lifetime NPVs than MD program graduates in all 47 specialties, with a median lifetime earning potential of $363,655 less; this represents 7% of the median Lifetime NPV for MD-PhD physicians (Table 1).

**Figure 1:**
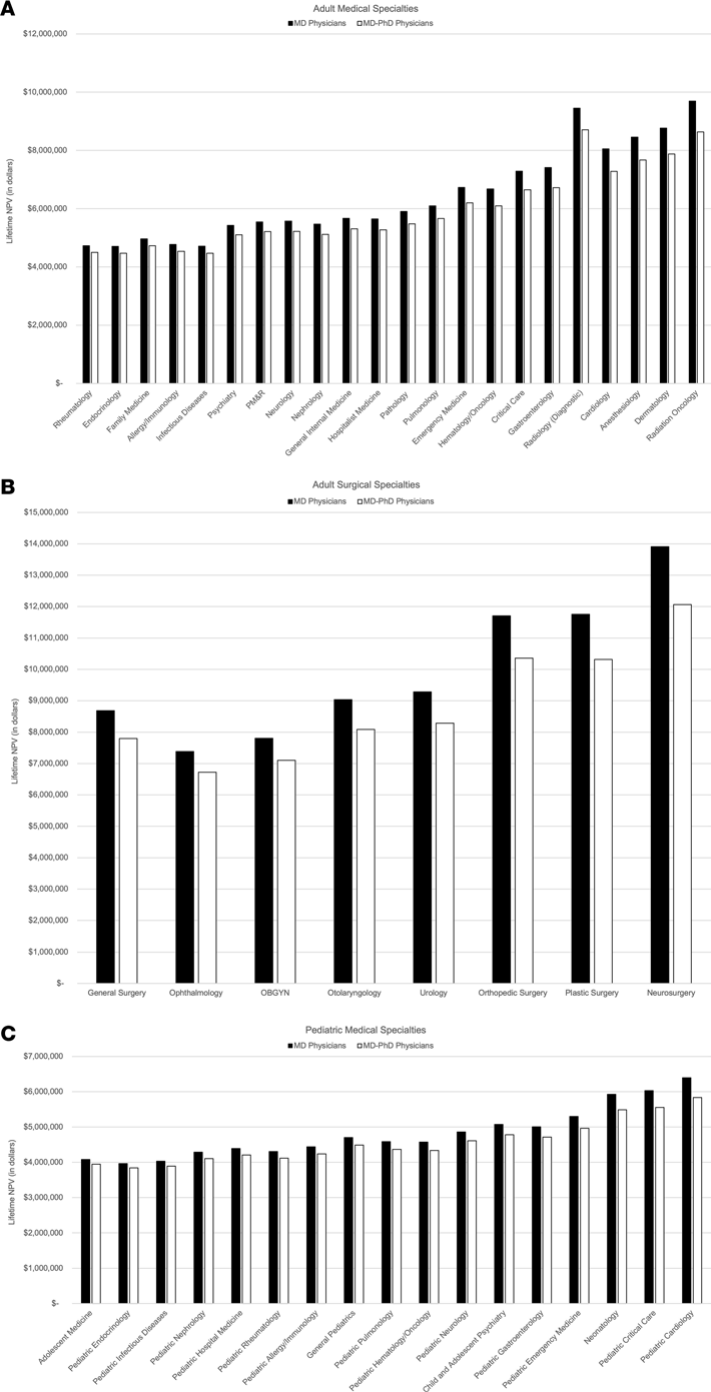
Lifetime Net Present Value for MD physicians compared with MD-PhD physicians. (**A**) Adult medical, (**B**) adult surgical, and (**C**) pediatric medical specialties. Specialties are ordered by size of difference in Lifetime Net Present Value between MD and MD-PhD physicians. PM&R, physical medicine and rehabilitation.

Notably, however, the MD-PhD pathway tended to be less financially disadvantageous for specialties with longer training durations or lower after-training salaries. These specialties were, in general, more outpatient based and less procedure oriented (Figure 1, A–C). For example, MD-PhD physicians practicing in pediatric endocrinology had a Lifetime NPV only $121,139 lower than MD physicians working in this same field. This difference represents approximately 3% of total Lifetime NPV for MD-PhD pediatric endocrinologists. MD-PhD neurosurgeons, however, had a Lifetime NPV $1,839,636 lower than MD neurosurgeons, representing 15% of total Lifetime NPV for MD-PhD neurosurgeons.

### Impact of interest rates on Lifetime NPV.

The NPV calculation discounts future earnings to address the concept that money available now is worth more than the same amount would be in the future since it can be invested to earn a return. When interest rates increase, the discount rate increases, and the value of future earnings decreases. We included a sensitivity analysis to capture the impact of different interest rates on our models. At a discount rate of 2.25%, which is what we used for our primary modeling based on historical trends (11), median Lifetime NPV was $5,643,495 for MD physicians and $5,274,820 for MD-PhD physicians (Table 1). However, at a discount rate of 5%, median Lifetime NPV decreased to $3,051,880 for MD physicians and $2,774,139 for MD-PhD physicians (Supplemental Table 1; supplemental material available online with this article; https://doi.org/10.1172/jci.insight.183476DS1). Additionally, the difference between Lifetime NPV for MD and MD-PhD physicians was modestly higher at a discount rate of 2.25% ($363,655) compared with a discount rate of 5% ($277,741) (Supplemental Table 1).

### Sex differences in lifetime earning potential.

When Lifetime NPV was estimated separately for each sex, we found that the MD-PhD pathway tended to be less financially disadvantageous for women than for men, primarily because women are on average paid less than men (Supplemental Table 2). For example, male MD-PhD–trained neurosurgeons had a Lifetime NPV that was $1,865,242 (15%) less than that of male MD-trained neurosurgeons, but female MD-PhD–trained neurosurgeons had a Lifetime NPV that was $1,087,524 (12%) less than that of female MD-trained neurosurgeons. Similarly, for pediatric nephrology, MD-PhD–trained men had a Lifetime NPV $235,419 (6%) less than MD-trained men while MD-PhD–trained women had a Lifetime NPV $79,119 (2%) less than MD-trained women.

### Impact of delays in training for MD-PhD physicians.

Additional delays before earning a full salary for MD-PhD physicians occur for reasons such as a premedical gap year or time spent as an instructor after completing postgraduate training but before attaining a faculty appointment as an assistant professor. These delays were associated with a further reduction in earning potential for MD-PhD physicians (Table 2). The financial impact of additional delays was larger for the higher earning specialties — for example, spending a year as an instructor would reduce Lifetime NPV for MD-PhD–trained neurosurgeons by $2,302,222 (19%). In contrast, spending a year as an instructor for MD-PhD–trained infectious diseases physicians would reduce Lifetime NPV by $317,425 (7%). The gaps after college for MD-PhD program trainees are frequently longer than 1 year, as are the delays between completing board requirements for clinical training and becoming an assistant professor. Although not modeled here, each additional year of delay would have a further negative effect on Lifetime NPV.

### Relationship between lifetime earning potential, research effort, and MD-PhD workforce distribution.

**Figure 2:**
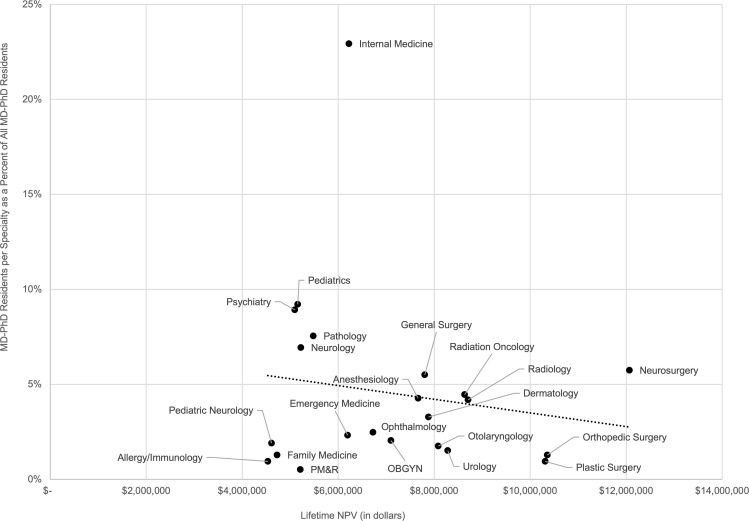
Association of Lifetime Net Present Value for MD-PhD physicians with the number of MD-PhD residents training in each specialty as a percentage of all MD-PhD residents.

We used bivariate linear regression analysis to examine the relationship between lifetime earning potential and MD-PhD workforce distribution. We found that the Lifetime NPV of a given specialty was not significantly associated with the percentage of MD-PhD residents choosing that specialty (–0.04% MD-PhDs/$100,000 Lifetime NPV, *P* = 0.49, 95% CI –0.14% to 0.07%; Figure 2 and Supplemental Table 3). In other words, on average, MD-PhD program alumni do not appear to be basing career specialty choices on future income.

**Figure 3:**
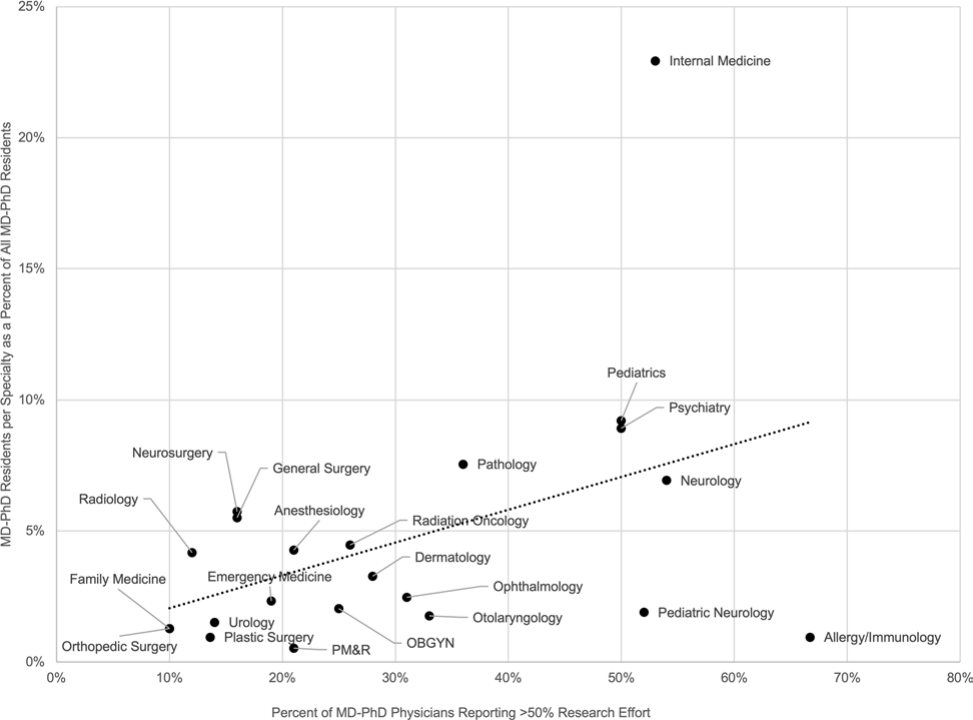
Association of the percentage of MD-PhD physicians reporting ≥50% research effort with the number of MD-PhD residents training in each specialty as a percentage of all MD-PhD residents.

However, the percentage of MD-PhD physicians in a specialty reporting ≥50% research effort was significantly associated with the percentage of all MD-PhD residents choosing that specialty (+12.5% MD-PhDs/1% increase in MD-PhDs reporting ≥50% research effort, *P* = 0.041, 95% CI 0.58% to 24.50%; Figure 3 and Supplemental Table 3). Stated differently, the specialties where MD-PhD physicians were more likely to report high research effort tended to be those with more MD-PhD physicians. For example, 7% of MD-PhD residents specialized in neurology, and 54% of MD-PhD neurologists reported spending ≥50% of their time engaged in research. However, only 1% of MD-PhD residents specialized in orthopedic surgery, and only 10% of MD-PhD orthopedic surgeons reported ≥50% research effort. Notably, we also ran this analysis excluding internal medicine as a potential outlier, as this specialty attracts the highest percentage of MD-PhD (and MD) residents. The relationship was no longer statistically significant (*P* value 0.11, 95% CI –0.014 to 0.131), suggesting that internal medicine was primarily driving the association.

**Figure 4:**
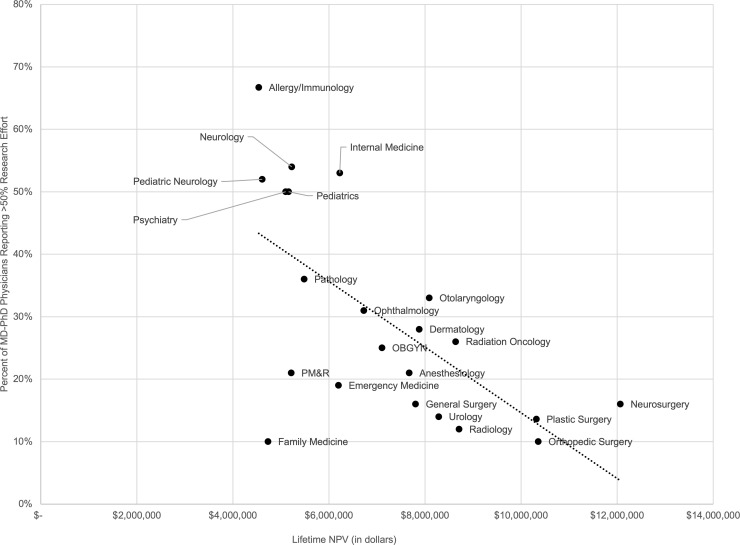
Association of Lifetime Net Present Value for MD-PhD physicians with the percentage of MD-PhD physicians reporting ≥50% research effort by specialty.

Finally, the percentage of MD-PhD physicians reporting ≥50% research effort was significantly negatively correlated with lifetime earning potential (–0.5% MD-PhDs reporting ≥50% research effort/$100,000 Lifetime NPV, *P* = 0.001, 95% CI –0.82% to –0.23%; Figure 4 and Supplemental Table 3). Stated differently, the research effort reported by MD-PhD program alumni in academia tended to be lower in higher earning specialties. For example, in radiology, with a Lifetime NPV of $8,706,823, 12% of MD-PhDs reported ≥50% research effort while in pediatric neurology, with a Lifetime NPV of $4,609,595, 52% of MD-PhDs reported ≥50% research effort.

## Discussion

When MD-PhD programs were originally established in the 1950s and 1960s, the intent was to provide future physician-scientists rigorous research and clinical training, with the goal of preparing graduates for research-focused careers (1). In light of the facts that physicians in full-time clinical practice in the United States can command higher salaries than research-focused physicians and that indebtedness could be an obstacle to a research career, tuition remission and annual stipends became commonplace, especially in MD-PhD training programs that received NIH National Institute of General Medical Sciences (NIGMS) support. Over the subsequent decades, the average time to graduate from MD-PhD programs has grown from approximately 6.7 years to just over 8 years. In addition, gaps of 1 or more years between college and medical school are more common for MD-PhD students than for MD students (12). Furthermore, the time from graduation to first faculty appointment has increased in most cases (13, 14). While the reasons for these changes are multifactorial, they have greatly prolonged the total time spent in training and have raised the average age at first faculty appointment and at first approval of an NIH R01 grant for MD-PhD–trained physician-scientists (13). The increased time until earning a full faculty-level salary represents forgone income. Here we asked, first, whether there is a substantial gap in lifetime earning potential for MD-PhD physician-scientists in academia compared with their MD colleagues within the same specialty and, second, whether there is evidence that an earnings gap between physician-scientists and clinicians is associated with decisions about choice of clinical specialty and research effort in academia.

While there was wide variability in the financial returns of medical training related to specialty of choice, in every specialty analyzed, our results showed that pursuing an MD-PhD degree led to a lower lifetime earning potential than pursuing an MD degree alone. This was especially evident for procedure-oriented specialties, particularly the surgical specialties. For more outpatient-based and less procedure-oriented specialties, particularly the pediatric specialties, the impact on lifetime earning potential was smaller, although the directionality was the same. This reflects the fact that non-procedure-oriented specialties generally earn less than procedure-oriented specialties, so additional years of training in these lower earning fields decreases lifetime earning potential by less than additional years of training in higher earning fields. Any further delay, such as a gap year before medical school or time spent as an instructor, added to the relative negative financial impact of MD-PhD training.

However, despite these results, we did not find that lifetime earning potential was significantly associated with the pattern of specialization of MD-PhD physicians. Some of the lowest earning specialties, such as pediatrics, neurology, and pathology, attract many MD-PhD physicians. Conversely, some of the highest earning specialties, including orthopedic surgery and radiology, have relatively fewer MD-PhD physicians. Instead, the rates of specialization were associated with the proportion of MD-PhD physicians who reported spending ≥50% of their time dedicated to research. While this finding was primarily driven by the large percentage of MD-PhD residents who specialize in internal medicine, it suggests that MD-PhD trainees’ career decisions may be motivated by the desire to have research participation be a core component of their careers. Role models are important when trainees make career choices. Mentorship opportunities may be more accessible in specialties with a higher proportion of MD-PhD physicians, especially those who are primarily researchers. It also may be the case that in fields in which clinical work is highly remunerative, such as the surgical specialties, MD-PhD physicians are discouraged from pursuing less well-compensated research activities, which in turn discourages MD-PhD trainees interested in research-focused careers from applying for postgraduate training positions in these specialties. This is supported by our finding that reported research effort was inversely correlated to lifetime earning potential. Finally, while MD-PhDs consistently had lower lifetime earning potential than MD physicians across all specialties, the differences between MD-PhD physicians and MD physicians within a specialty were typically much smaller than the differences in lifetime earning potential between specialties regardless of training pathway. Thus, it may be the case that earning potential influences trainees’ decisions regarding clinical specialty, as we have previously shown in the pediatric subspecialties (9), but not degree type. This difference also may reflect the fact that a trainee decides on degree type prior to entering medical school compared with specialty selection, which typically is made closer to the end of medical school. The fact that MD-PhD trainees appear to choose medical specialties based on the likelihood they will be able to pursue research suggests that lifetime earning potential is not a primary motivation for specialty choice for most MD-PhD graduates.

These findings have implications for the development and maintenance of the physician-scientist workforce. Overall, the MD-PhD training pathway has been successful in training physician-scientists. The 2015 National MD-PhD program outcomes study found that 77% of MD-PhD program graduates spent some time doing research (13). The NIH Physician-Scientist Workforce Advisory Group reported that MD-PhD program graduates were more likely to apply for and receive NIH training awards and research grants than their MD-only counterparts (2). However, the longer training time and the negative financial impacts associated with this may discourage candidates from applying to MD-PhD programs. A survey of research-oriented medical trainees including both MD-PhD and MD students found that the MD students were significantly more likely to report loan repayment as an obstacle to career advancement, whereas MD-PhD students were more likely to report undercompensation (15). Moreover, while many studies focus on factors that influence MD-PhD candidates’ decisions to enter training, few studies assess the factors that dissuade potential candidates from applying to MD-PhD programs in the first place. There may be a subset of medical students for whom MD-PhD training is not achievable financially. Shortening the length of MD-PhD training and, in particular, the time to independence as a researcher would lessen the negative relative financial impact of MD-PhD training and has been a consistent focus of efforts to increase the physician-scientist workforce (16). Enhanced earnings for MD-PhD trainees, such as higher stipends during medical school or higher salaries during residency/fellowship, also would mitigate the negative impact of prolonged training by further reducing the financial stresses of these periods.

Women are increasingly entering MD-PhD training programs (17). Interestingly, we found that MD-PhD training was less financially disadvantageous for women than for men, partly because women have lower after-training salaries across all specialties (8). Until very recently, women were underrepresented in MD-PhD programs, and they are still considerably underrepresented in the physician-scientist workforce, with fewer women obtaining full-time faculty appointments and NIH grants or moving successfully from mentored to independent NIH grants (13, 17). Our finding that the financial impact of MD-PhD training varies by sex may be useful to inform future recruitment and funding strategies both to encourage women to apply to MD-PhD training programs and to support women once in the physician-scientist workforce. MD-PhD physicians, particularly women, are more likely to have children while still in training, and personal/professional integration is a significant barrier to retention of MD-PhD program graduates (15). Thus, medical schools and residency/fellowship programs might consider allowing more flexibility surrounding parental leave and providing free or subsidized childcare. The additional expenses of such programs should be evaluated against the loss of institutional and taxpayer dollars invested in a trainee who leaves the workforce for a more flexible career because of childcare obligations.

### Limitations of the study.

Our results are dependent upon the assumptions inherent to our models, which include those regarding training length, timing of academic promotion, and rates of debt repayment. In general, our models likely underestimate the differences in lifetime earning potential between MD-PhD and MD training pathways. For example, in our models of additional delays for MD-PhD physicians, we assumed an instructor salary rather than a postdoctoral fellow salary, which is much lower and which would increase the already-noted differences between training pathways. We also assumed that MD-PhD physicians did not have educational debt; factoring in educational debt for MD-PhD physicians would also increase the differences between the 2 pathways. Furthermore, because the Association of American Medical Colleges (AAMC) annual Medical School Faculty Salary Survey Report does not provide data disaggregated by degree type, we were not able to assess whether after-training salaries for MD-PhD and MD graduates were indeed equivalent. The same values were assigned to both, which may not be the case, as research-focused faculty may earn lower salaries than clinically oriented faculty. The NIH sets a cap for the direct salary paid to an individual under a grant, which is typically lower than full-time clinical salaries: in 2020, the year upon which our salary data are based, this cap was $197,300 (18). While some departments supplement salaries for research time to match clinical salaries, those that do not exacerbate the lower lifetime earning potential for MD-PhD physicians. However, for MD-PhDs who end up in full-time clinical practice, we would expect salaries would be equal to those of MD physicians. We also did not evaluate the financial impact on MD-PhD physicians working in industry (~7%), which is considered a desirable outcome of MD-PhD training, or nonacademic full-time clinical practice (~24%), which is not considered a desirable outcome of MD-PhD training (13, 14).

The AAMC faculty salary data are insufficient to allow for modeling disaggregated by race and ethnicity. Overall, the available evidence suggests that underrepresented minorities often have higher educational debt burdens and lower after-training salaries (19, 20). As a group, members of underrepresented minorities in MD-PhD programs have a longer average time to degree (8.4 years versus 8.2 years in the 2005 to 2014 graduation cohorts) and are less likely to obtain NIH grants following graduation (17). Thus, the financial impact of MD-PhD training is potentially more onerous for members of underrepresented minorities in medicine and is an important area for future study.

Furthermore, there are likely regional differences in lifetime earning potential. The AAMC does provide salary data across different US regions; this is an important area of future study that would benefit from additional modeling to capture variability in cost of living and salary data disaggregated by degree type and thus is beyond the scope of the current study.

We also note that having an MD-PhD degree in the NIH research database does not necessarily mean that the individual graduated from an integrated MD-PhD program, although most MD-PhDs probably did. This issue also clouds the evaluation of NIH funding data, as was noted in the 2014 NIH Physician-Scientist Workforce (PSW) Working Group Report (2). Finally, the data from the MD-PhD Outcomes Study were collected up until 2015, and there may be more recent trends in specialization patterns that are not captured in our data. Future studies that periodically reevaluate the relationship between lifetime earning potential and MD-PhD workforce distribution are necessary.

### Conclusions.

This study demonstrates that there is wide variability in the financial returns of MD-PhD training related to clinical specialty choice and that these returns are lower than those of MDs training in the same specialty. Reported research effort was negatively correlated with lifetime earning potential: MD-PhD program alumni in disciplines with the highest earning potential spent less time on research. However, the percentage of MD-PhD physicians who chose each specialty was not correlated with lifetime earning potential. Instead, MD-PhD alumni tended to choose specialties that afforded the opportunity to have more research time. MD-PhD physicians pursue their training for a myriad of reasons, including greater intellectual stimulation, the possibility to have a profound impact on human health through the results of research, a desire to work with specific patient populations, interest in particular diseases or organ systems, and greater opportunities for teaching. Thus, it is hardly surprising that eventual income (how much and how soon) might be a less important career-shaping factor. Supporting a robust physician-scientist workforce should be a priority among institutional, governmental, and industry stakeholders. The financial impact of MD-PhD training as well as the ultimate ability of MD-PhD physicians to successfully engage in research-oriented careers should be considered when developing strategies to increase recruitment and retention of physician-scientists.

## Methods

### Data sources.

For this study, we obtained information on training stipends, specialty-specific mean compensation, and educational debt for the academic year of July 2020 to June 2021.

We assumed that MD physicians did not have any income while in medical school. To our knowledge, a national average of the stipends provided for MD-PhD trainees has not been published. A review of programs that provide stipend information on their websites suggests that annual stipends range from $24,000 to $48,000. Thus, we assumed a stipend of $36,000 per year for MD-PhD trainees during all 8 years of their medical school and PhD training.

To estimate residency and fellowship compensation for each postgraduate year (PGY) from PGY1–7, we used the annual AAMC Survey of Resident/Fellow Stipends and Benefits (21). Salaries during residency and fellowship are equal across specialties and for both MD and MD-PhD trainees.

For postfellowship specialty-specific compensation estimates by academic rank, we utilized data from the AAMC’s annual Faculty Salary Report (22). AAMC faculty compensation data represent the mean fixed/contractual salary component of total compensation plus the supplemental earnings components of total compensation (medical practice supplement, bonus/incentive pay, and known uncontrolled outside earnings), before taxes and retirement/fringe benefits, of full-time faculty affiliated with Liaison Committee on Medical Education–accredited medical schools. The 2020–2021 survey reflected data from 152 medical schools. The AAMC Faculty Salary Survey does not differentiate average salary between MD and MD-PhD physicians, and thus we assumed the same salary by years at rank for both groups.

We obtained mean educational debt from the AAMC 2021 Debt Fact Card (23). For our initial model for MD physicians, we assumed that loan repayment was deferred during residency and fellowship and then was repaid over 25 years and that the accrued interest was capitalized once training was completed. We used an interest rate of 5.28% based on the rates of federal Stafford educational loans for 2020–2021 (23). The majority of MD-PhD physicians have no debt or relatively small debt burdens (24), and so, for our analysis, we assumed that MD-PhD physicians had no educational debt.

Data regarding MD-PhD physician workforce distribution, including the numbers of graduates who completed residency or fellowship training in a given specialty and the percentage research effort reported by MD-PhD physicians by specialty, were obtained from published data from the National MD-PhD Program Outcomes Study (13, 14). That study included survey data from 3,025 MD-PhD program alumni working in academia.

### Estimation of lifetime earning potential.

As described in our prior reports (4–6, 9), we used cross-sectional compensation-by-rank data to generate annual net income streams for academic physicians over a working lifetime. For our initial analysis, we assumed no time was taken off between high school, college, medical school, residency, fellowship, and employment. For MD physicians, we assumed a medical school duration of 4 years, and for MD-PhD physicians, we assumed a medical school and PhD training period of 8 years (13). We obtained typical training lengths for the adult specialties from the American College of Physicians and for the pediatric specialties from the American Board of Pediatrics (25, 26). We assumed that, after completing residency and fellowship training, both MD and MD-PhD physicians worked as assistant professors for 7 years, then as associate professors for 7 years, and then as full professors for the remainder of their careers (27, 28). We assumed a retirement age of 65 based on data from a systematic review that demonstrated the majority of physicians, including those in academic practice, retire between ages 60 and 69 (29).

To compare overall lifetime earning potential between MD and MD-PhD physicians, we employed the concept of NPV, which is a standard financial technique used to analyze the value of different income streams over time (4, 10). NPV addresses the concept that income obtained today is more valuable than future income, because today’s income can be invested to yield an immediate return. NPV analysis is used in finance to evaluate the value of potential investments by discounting future income back to the present at a constant discount (or interest) rate. A higher NPV indicates a higher current value of future net income streams.

The formula for NPV is:
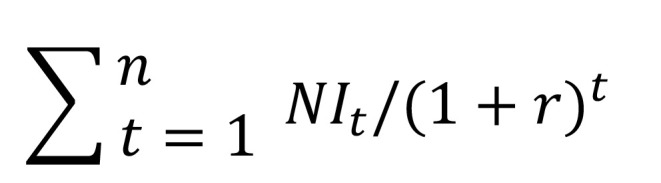


where *NI* is annual net income, which we defined as annual compensation less annual debt repayment costs (10). The formula takes the sum of the annual net incomes over time (from *t* = 1 to *n* years) and discounts them back to the present at a discount rate (*r*), which was set at 2.25% based on the discount rate in February of 2020, prior to the COVID-19 pandemic, during which interest rates fell to near-historic lows (11). We also included a sensitivity analysis where we used a discount rate of 5.0% to capture how fluctuating interest rates impacted our modeling.

We defined the Lifetime NPV as the present value of the net income generated from a career in a specialty over a working lifetime. The Lifetime NPV is an estimate of a physician’s lifetime earning potential and represents the present value of the financial returns that a matriculating medical student might expect from the investment of entering either an MD or MD-PhD program.

### Sex differences in lifetime earning potential.

We previously demonstrated that female physicians have, in general, lower earning potential than male physicians (8). To further evaluate this disparity, we compared the Lifetime NPV for MD physicians versus MD-PhD physicians for each specialty disaggregated by sex.

### Sensitivity analyses of delays in training for MD-PhD physicians.

The first set of sensitivity analyses focused on modeling the impact of additional delays in the MD-PhD training pathway.

Recent data suggest that MD-PhD physicians are more likely than MD physicians to take training gaps of 1 to 2 years prior to medical school and that this trend has increased over time (12). For this sensitivity analysis, we recalculated the Lifetime NPV for MD-PhD physicians by including a 1-year delay in starting medical school, effectively shortening their careers after training by 1 year.

Additionally, many MD-PhD physicians spend time as postdoctoral fellows or instructors following completion of residency or fellowship training and before getting a faculty appointment as an assistant professor. Data from the National MD-PhD Program Outcomes Study found the median time to first academic faculty position (instructor or assistant professor) after graduation was 6 years (13). Based on this data, we modeled an additional scenario for MD-PhD physicians reflecting a 1-year delay in obtaining an assistant professor position after training completion, during which time they earned an instructor salary rather than an assistant professor salary.

### Relationship between lifetime earning potential, research effort, and MD-PhD workforce distribution.

We evaluated the relationship between lifetime earning potential, reported research effort, and MD-PhD workforce distribution using bivariate linear regression analyses. We obtained data regarding the percentage of MD-PhD physicians reporting that they engaged in research activities for ≥50% of their full-time effort by specialty from the National MD-PhD Outcomes Study. Using data from the AAMC Report on Residents (30), we characterized MD-PhD physician workforce distribution by calculating the number of MD-PhD residents per specialty as a percentage of all MD-PhD residents.

### Statistics.

For our statistical analysis evaluating the relationship between lifetime earning potential, reported research effort, and MD-PhD workforce distribution using bivariate linear regression analyses, we used a *P* value of less than 0.05 to determine statistical significance.

### Study approval.

Because we used publicly available, aggregated, and deidentified data, this study did not meet the criteria for human research and did not require institutional review board approval.

### Data availability.

Compensation and debt data used in this study are available through the AAMC. Data regarding the MD-PhD physician workforce are available through the National MD-PhD Outcomes Study. Data represented in figures are available in the supplemental Supporting Data Values file accompanying this manuscript online.

## Author contributions

EC was primarily responsible for study design, data acquisition and analysis, and manuscript writing. JR, MHA, LFB, and HKS were responsible for study design, data analysis, and manuscript writing and editing.

## Supplementary Material

Supplemental data

Supporting data values

## Figures and Tables

**Table 1:**
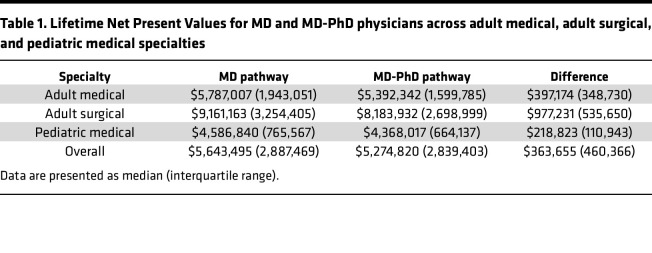
Lifetime Net Present Values for MD and MD-PhD physicians across adult medical, adult surgical, and pediatric medical specialties

**Table 2:**
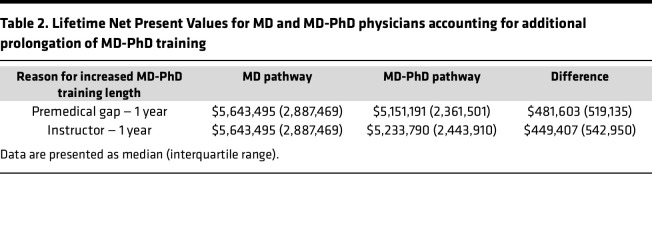
Lifetime Net Present Values for MD and MD-PhD physicians accounting for additional prolongation of MD-PhD training

## Version 1 12/20/2024

Electronic publication
