# Supplementary material for: The financial impact of MD-PhD training compared with MD training for academic physicians
Source: JCI Insight. 2024 Dec 20;9(24):e183476. doi: 10.1172/jci.insight.183476 (PMC11665557; doi:10.1172/jci.insight.183476)
Supplement: Supplemental data [file jciinsight-9-183476-s259.pdf]

The financial impact of MD-PhD training compared with MD training for academic physicians

Eva Catenaccio,[1] Jonathan Rochlin,[2] Myles H. Akabas,[3] Lawrence F. Brass,[4] and Harold K. Simon[5]
1. Department of Neurology, Division of Pediatric Neurology, Perelman School of Medicine at the University of Pennsylvania and the Children's Hospital of Philadelphia, Philadelphia, Pennsylvania, USA. 2. Department of Emergency Medicine, Division of Pediatric Emergency Medicine, Maimonides Medical Center, Brooklyn, New York, USA. 3. Departments of Neuroscience and Medicine, Albert Einstein College of Medicine, Bronx, New York, USA. 4. Department of Medicine, Perelman School of Medicine at the University of Pennsylvania, Philadelphia, Pennsylvania, USA. 5. Departments of Pediatrics and Emergency Medicine, Emory University School of Medicine and Children's Healthcare of Atlanta, Atlanta, Georgia, USA.

Supplementary Table 1. Lifetime Net Present Values (NPV) for MD and MD-PhD physicians across adult medical, adult surgical, and pediatric medical specialties with a discount rate of five percent. Data are presented as medians (interquartile range).

Specialty	MD Pathway	MD-PhD Pathway	Difference
Adult Medical	$3,148,124 (979,585)	$2,842,919 (760,010)	$305,204 (228,168)
Adult Surgical	$4,858,491 (1,619,405)	$4,157,079 (1,238,258)	$701,413 (380,846)
Pediatric Medical	$2,429,077 (445,204)	$2,265,142 (354,872)	$163,935 (90,332)
Overall	$3,051,880 (1,565,303)	$2,774,139 (1,228,886)	$277,741 (341,362)

Supplementary Table 2. Lifetime Net Present Values (NPV) for MD and MD-PhD physicians across adult medical, adult surgical, and pediatric medical specialties disaggregated by gender. Data are presented as median (interquartile range).

Specialty	MD Pathway	MD-PhD Pathway	Difference
Adult Medical - Men	$5,938,690 (1,983,241)	$5,520,659 (1,639,111)	$418,031 (346,254)
Adult Medical - Women	$5,473,327 (1,830,825)	$5,140,648 (1,502,262)	$332,679 (329,385)
Adult Surgical - Men	$9,306,565 (3,548,342)	$8,315,364 (2,918,950)	$991,201 (624,058)
Adult Surgical - Women	$8,031,395 (1,914,440)	$7,247,272 (1,571,284)	$784,123 (334,538)
Pediatric Medical - Men	$4,719,248 (742,059)	$4,453,144 (623,728)	$258,033 (101,594)
Pediatric Medical - Women	$4,357,800 (774,803)	$4,206,616 (633,093)	$182,953 (141,710)

Supplementary Table 3. Detailed results of the linear regression analyses shown in Figures 2-4.

Dependent Variable	Independent Variable	N	F-statistic	R-squared	Regression Coefficient	P-value	95% CI LB	95% CI UB
MD-PhD Residents per Specialty as Percent of all MD-PhD Residents	Lifetime NPV in 100,000's of Dollars	22	0.49	0.0238	-0.00036	0.493	-0.0014355	0.0007155
MD-PhD Residents per Specialty as Percent of all MD-PhD Residents	Percent of MD-PhD Physicians Reporting ≥50% Research Effort	22	4.79	0.1931	0.1254032	0.041	0.0058442	0.2449622
Percent of MD-PhD Physicians Reporting ≥50% Research Effort	Lifetime NPV in 100,000's of Dollars	22	14.1	0.4135	-0.0052592	0.001	-0.0081804	-0.002338
